# Prognostic Role of Polyvascular Involvement in Patients with Symptomatic Peripheral Artery Disease

**DOI:** 10.3390/jcm12103410

**Published:** 2023-05-11

**Authors:** Luise Adam, Eva Strickler, Meisam K. Borozadi, Simone Bein, Arjola Bano, Taulant Muka, Heinz Drexel, Jörn F. Dopheide

**Affiliations:** 1 Division of Angiology, Swiss Cardiovascular Center, Inselspital, University Hospital Bern, University of Bern, 3010 Bern, Switzerland; 2 Institute of Primary Health Care (BIHAM), University of Bern, 3012 Bern, Switzerland; 3 Division of Angiology, Gefässzentrum Cantonal Hospital Baden, 5404 Baden, Switzerland; 4 Department of Cardiology, Inselspital, University Hospital Bern, University of Bern, 3010 Bern, Switzerland; 5 Division of Angiology, Cantonal Hospital Luzern, 6000 Luzern, Switzerland; 6 Department of Emergency Medicine, Inselspital, University Hospital of Bern, 3010 Bern, Switzerland; 7 ISPM, Institute of Social and Preventice Medicine, University of Bern, 3012 Bern, Switzerland; 8 Epistudia, 3011 Bern, Switzerland; 9 Vorarlberg Institute for Vascular Investigation and Treatment (VIVIT), 6900 Feldkirch, Austria; 10 Medical-Scientific Faculty, Private University of the Principality of Liechtenstein, 9495 Triesen, Principality of Liechtenstein; 11 Drexel University College of Medicine, Philadelphia, PA 19104, USA; 12 Department of Medicine I, Division of Cardiology, Angiology and Intensive Medical Care, University Hospital Jena, Friedrich-Schiller-University Jena, 07740 Jena, Germany

**Keywords:** polyvascular disease, peripheral artery disease, statin therapy

## Abstract

**Background:** Statin therapy is recommended for patients with peripheral artery disease (PAD). However, PAD patients with polyvascular (PV) extent remain threatened by an increased residual cardiovascular (CV) risk. **Purpose:** To investigate the association of prescribed statin therapy and mortality in PAD patients with or without PV extent. **Methods**: A single-center retrospective longitudinal observational study originating from a consecutive registry with 1380 symptomatic PAD patients over a mean observational time of 60 ± 32 months. The association of atherosclerotic extent and statin use (PAD, plus one additional region (CAD or CeVD, [+1 V]), +2 vascular regions (+CAD and CeVD [+2 V]) with the risk of all-cause mortality was evaluated using Cox proportional hazard models adjusted for potential confounding factors. **Results:** The mean age of the study’s participants was 72.0 ± 11.7 years, with 36% being female. PAD patients with PV extent [+1 V] and [+2 V] were older and suffered from diabetes, hypertension, or dyslipidemia more often; they, too, had more severely impaired kidney function (all *p* < 0.0001) compared to patients with PAD only. PAD patients with PV [+1 V] and [+2 V] received better statin medication and reached the recommended LDL-C target compared to PAD-only patients (*p* < 0.001). Despite better statin treatment, the rate of all-cause mortality was higher in PV patients than in PAD-only patients (PAD only: 13%; [+1 V]: 22%; [+2 V]: 35%; *p* < 0.0001). **Conclusion:** PV patients receive better statin therapy than PAD-only patients but nevertheless still have higher mortality rates. Future studies are needed to explore whether more aggressive LDL-lowering treatment for PAD patients may be translated into better prognosis.

## 1. Introduction

At present, cardiovascular disease still takes the highest death toll worldwide. Proceeding from this notion, efforts to reduce ischemic events and improve cardiovascular outcomes have been the driving force behind cardiovascular research in recent decades.

A growing body of evidence has identified a malignant phenotype defined as a symptomatic atherosclerotic disease in more than one territory (e.g., coronary, peripheral, cerebrovascular) and called polyvascular disease (PVD) [1].

Among single atherosclerotic locations, peripheral artery disease (PAD) has the highest cardiovascular risk, even higher than in patients with coronary artery disease (CAD) [2,3,4,5]. This might be due to dyslipidemia and the increased inflammatory state of the peripheral extent, as has been demonstrated by levels of C-reactive protein, which are higher in PAD than in coronary patients as well as the high co-prevalence of diabetes in PAD patients and, likely, other genetic factors [6,7,8]. A classic concept is that in PAD patients, a spread to other vascular beds is likely and that all patients with PAD essentially have coronary or cerebrovascular artery disease (CAD or CeVD, respectively), known or unknown [9].

Statin therapy is an established tool to decrease cardiovascular events and deaths in patients with established cardiovascular disease [10,11]. Even though PAD patients have a very high cardiovascular risk and there is solid evidence supporting the use of lipid-lowering therapy in secondary prevention, previous studies have reported issues and challenges in the attainment of LDL-C targets [12].

It remains unclear whether statin therapy is prescribed following the guidelines in polyvascular patients and if statin prescription has an influence on cardiovascular outcomes in polyvascular patients regarding their extent of polyvascular affection.

In the present study, we compare the outcome of statin-treated patients with PAD only to those with polyvascular disease.

## 2. Methods

### 2.1. Patients and Study Design

This study is a retrospective longitudinal observational study from a, single-center consecutive registry of patients with symptomatic PAD (Fontaine Stage II, III, IV) undergoing endovascular therapy. Patients were referred to our tertiary reference center from 2009 to 2019. The local ethics committee approved the use of patient data for clinical studies.

#### 2.1.1. Inclusion Criteria

Patients were eligible if they (i) were undergoing their first intervention for symptomatic PAD, (ii) were older than 40 years, (iii) had a baseline fasting lipid profile, (iv) and LDL-C was calculated by the Friedewald formula if triglycerides were ≤4.5 mmol/L [13].

#### 2.1.2. Exclusion Criteria

Exclusion criteria were non-atherosclerotic disease as underlying cause for peripheral arterial disease, such as acute or embolic occlusions and vasculitis. All patients undergoing repeat peripheral arterial intervention were excluded. Patients without available lipid profiles or TG > 4.5 mmol/L were excluded. A patient’s formal dissent to use clinical data for research also led to exclusion.

### 2.2. Assessment and Definition of Outcomes

At the time of study inclusion, prior to the first peripheral intervention, baseline characteristics such as the cardiovascular risk profile, atherosclerotic comorbidities, and medication, including statin therapy and dosage, as well as laboratory values, including kidney function and lipid profile, were recorded. Patients without any clinically apparent manifestation of CAD or CeVD were considered to have PAD only. All patients suffering from PAD and any clinically apparent other atherosclerotic manifestation in a vascular bed (CAD and or CeVD) were considered to be suffering from polyvascular atherosclerotic disease (PAD, +1 vascular region (+/−CAD or CeVD) (+1 V) or +2 vascular regions (+CAD and CeVD) (+2 V)) [14].

Survival status was assessed by linking with the national mortality record. Information about the diagnosis was obtained based on the International Statistical Classification of Diseases and Related Health Problems 10th version ICD through patient’s charts. Primary endpoint was all-cause mortality, defined as death from any reason, including cardiovascular death. To avoid uncertainties and biases in our primary analysis, we a priori did not include endpoints needing an adjudication by investigators and thus confined the primary analyses of endpoints to a fatal one.

Secondary endpoints were cardiovascular events, including major adverse cardiovascular events (MACE) and major adverse limb events (MALE). MACE was defined as a composite of cardiovascular death, ischemic stroke, or myocardial infarction [15]. MALE was defined as the first occurrence of acute limb ischemia (ALI), major amputation, and/or lower extremity revascularization [16].

### 2.3. Definition of Statin Treatment

Statin therapy at baseline was classified as either high, moderate, or low intensity. High-intensity statin therapy was defined as treatment with Atorvastatin 40–80 mg or Rosuvastatin 20–40 mg. Atorvastatin 10–20 mg, Fluvastatin 40 mg twice daily, Pravastatin 40–80 mg, Rosuvastatin 5–10 mg, and Simvastatin 20–40 mg were considered to be moderate-intensity statin treatment. Fluvastatin 20–40 mg, Pravastatin 10–20 mg, and Simvastatin 10 mg were considered low-intensity treatment [17,18]. The mean prescribed statin dosage was normalized to simvastatin 40 mg [18].

Lipid-lowering targets were assessed according to ESC guidelines with LDL-C first <1.8 mmol/L and <1.4 mmol/L in order to account for the change in guidelines in 2019 [11,19].

Assessment of covariates was performed at baseline using patient charts and prior information from their treating general practitioner for diagnosis of arterial hypertension and diabetes. Pre-interventional blood analyses were performed systematically, assessing serum creatinine, HbA1c, and the lipid profile (total cholesterol, HDL-C, and Triglycerides). LDL-C was calculated using the Friedewald formula [13]. Estimated Glomerular Filtration Rate (eGFR) was calculated using the CKD-EPI formula [20].

### 2.4. Statistical Analysis

Categorical data are shown as absolute numbers and percentages (%). Continuous variables are shown using mean ± standard deviation (SD) for variables with normal distribution. For variables with skewed distribution, median and interquartile ranges are provided. Normal distribution of the variables was investigated by visual inspection of the histogram. Natural log transformation was applied to the variables that were considered skewed for the Cox regression analysis to account for the potential effect of outliers. Subgroup differences for two or three groups were compared using the chi-2-test. For comparison of two groups with continuous variables, we used the Mann–Whitney–Wilcoxon test. For three-group comparison, we used the Kruskal–Wallis test. Due to the retrospective study design, we decided to focus our analyses on overall mortality, being the most robust endpoint and least likely to be biased. Survival data for the secondary endpoints are shown using Kaplan–Meier curves. Association of polyvascular disease (+1 V, +2 V) and PAD only with overall mortality in the overall population and separate by males and females was assessed using a Cox proportional hazard model; hazard ratios (HR) and 95% confidence intervals are reported (95% CI). We first built a model adjusted for age and sex (model 1), and then, based on literature, built a second model additionally adjusting for potential confounders and traditional cardiovascular risk factors, including smoking status, LDL-C levels, statin therapy, hypertension, creatinine levels and diabetes (model 2). The proportional hazards assumption was checked using Schoenfeld residuals. This approach showed no violation of the proportionality assumption (*p* > 0.005 for models 1 and 2, Figure S2a,b). In order to explore whether the association between PAD patient groups and mortality would differ by smoking status and diabetes, an interaction term was tested in the model. *p*-values < 0.05 were considered significant.

All statistical analyses were performed using GraphPad Prism statistical software package, version 8.4.3 (GraphPad Software, San Diego, CA, USA) and Stata 17.0 StataCorp. 2021. (Stata Statistical Software: Release 17. College Station, TX, USA: StataCorp LLC.).

## 3. Results

### 3.1. Patients Characteristics

Our prospective registry from 2010–2020 included 1939 patients undergoing their first endovascular peripheral arterial revascularization. A total of 491 patients were excluded based on the above-mentioned exclusion criteria. For 71 patients, we did not have any information on follow-up (Figure S1).

This study includes 1377 PAD patients from our single-center registry (36.4% female), mean age of 72.0 ± 11.7 years (Figure S1). Most patients had PAD only (*n* = 688, 50%), while 552 (40%) had PAD + 1 V and 137 (10.0%) had PAD + 2 V. Patients with polyvascular disease were significantly older (*p*< 0.0001) and were more often male (*p* < 0.01) (Table 1). Polyvascular patients had a more extended cardiovascular risk profile than patients with PAD only. Hypertension (*p* < 0.001), hyperlipoproteinemia (*p* < 0.001) and diabetes (*p* < 0.001) were more prevalent in PAD + 1 V and PAD + 2 V. In contrast, active smoking was more common in PAD-only patients (*p* < 0.0001). All baseline characteristics are presented in Table 1.

### 3.2. Lipid Profile and Statin Therapy at Baseline

**Figure 1:**
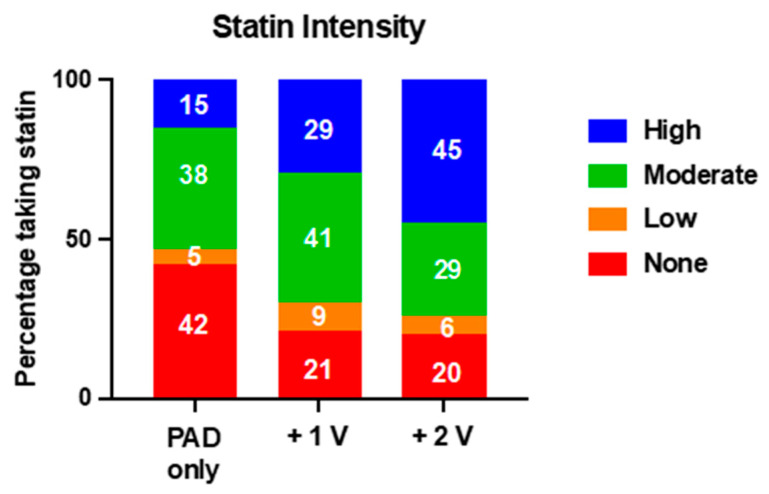
Relative distribution of statin treatment and intensity in PAD-only and polyvascular patients.

**Figure 2:**
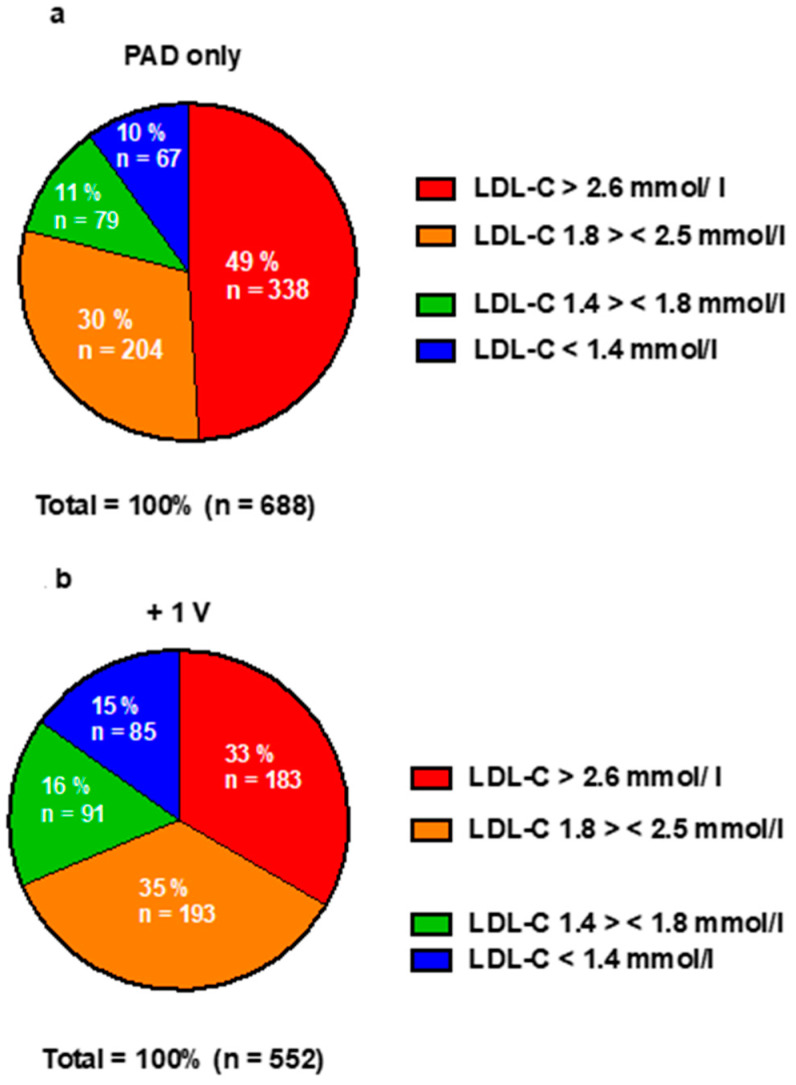

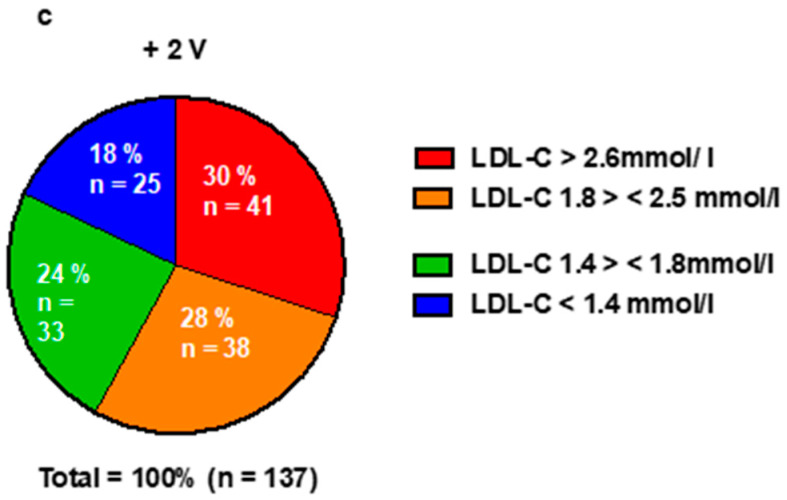
(**a**) LDL-C distribution in PAD-only patients. (**b**) LDL-C distribution in PAD +1 V patients. (**c**) LDL-C distribution in PAD +2 V patients.

A total of 949 (79.2%) patients were prescribed statins at baseline in the entire patient sample. Statin treatment at baseline was established in 408 (59.2%) patients for PAD only, 437 (79.3%) patients for the +1 V group, and 111 (81.0%) patients for the +2 V group. In the PAD-only group, statin treatment consisted of mainly moderate-intensity treatment, while the percentage of high-intensity treatment was higher with increasing polyvascular affection (15% in PAD-only patients, 29% +1 V, 45% +2 V, Figure 1). LDL-C goal (<1.8 mmol/L) was reached in 11% of PAD-only patients, while it was reached in 16% for +1 V and 24% for +2 V. Similar findings were seen when a target <1.4 mmol/L was applied (10% for PAD only, 15% for +1 V, 18% for +2 V). Further LDL-C distribution stratified by regional vascular affection is shown in Figure 2A–C as well as in Table S2.

### 3.3. Lipid Profile at Follow-Up

**Figure 3:**
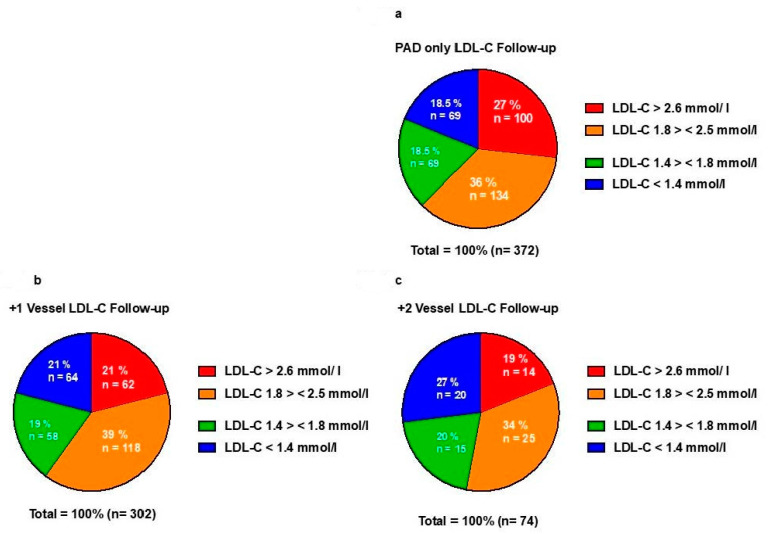
Relative distribution of statin treatment and intensity in PAD-only and polyvascular patients at Follow-Up. (**a**) LDL-C distribution in PAD-only patients at Follow-Up. (**b**) LDL-C distribution in PAD +1 V patients at Follow-Up. (**c**) LDL-C distribution in PAD +2 V patients at Follow-Up.

At the time of the event or last contact with the patient, (18.5%) of patients with PAD only met the LDL-C target of <1.4 mmol/ (18.5% for >1.4 mmol/L, <1.8 mmol/L). This goal was reached by 21% of the patients in +1 V (19.2% for >1.4 mmol/L, <1.8 mmol/L) and 27% of the patients in +2 V (20.3% for >1.4 mmol/L, <1.8 mmol/L) (Figure 3).

### 3.4. Assessment of Main Endpoints

The mean observational time was 59 ± 32 months. Over this period, 264 (19.2%) patients died, of which 100 (37.4%) were considered cardiovascular deaths, and 730 (50.1%) patients had cardiovascular events (151 (10.1%) MACE, 579 (42.1%) MALE).

There was a significant difference in all-cause mortality between the PAD-only group, +1 V and +2 V polyvascular patients over time (PAD only: 13%; [+1 V]: 22%; [+2 V]: 35%; *p* < 0.0001) (Figure S3).

Using multivariable Cox-regression analysis, adjusted for statin use, known cardiovascular risk factors, creatinine levels, and polyvascular disease, three-vessel atherosclerosis showed the highest hazard ratio for overall mortality (HR 2.83, 95%CI: 1.97–4.08, *p* < 0.001), and two vessel atherosclerosis the second highest hazard ratio (HR 1.84, 95%CI 1.39–2.45, *p* < 0.001). Among the confounding factors, age (HR 1.03, 95%CI: 1.01–1.04) and higher serum creatinine (HR 2.47, 95%CI: 1.94–3.15) was associated with increased risk of mortality. No association was observed between prior diagnosis of hypertension (HR 0.90, 95%CI: 0.59–1.40), sex (HR 0.87, 95%CI 0.65–1.17), smoking status (active smoking HR 0.90, 95%CI: 0.65–1.24, former smoking HR 0.76, 95%CI: 0.52–1.10) and mortality (Table 2). Stratified results by sex showed that the results were similar between men and women (Supplementary Table S1). In addition, interactions of PAD categories with sex, smoking status, and diabetes were not significant (*p*-values < 0.05).

Polyvascular patients were at higher risk for cardiovascular events than patients with PAD only. In the adjusted Cox model, patients in the +2 V group had the highest risk for cardiovascular events (+2 V HR 1.90, 95% CI: 1.10–3.30; +1 V 1.86, 95% CI 1.28–2.68) (Table S3) while none of the confounding factors were significantly associated with cardiovascular events.

## 4. Discussion

In this large observational, retrospective study of 1377 patients with symptomatic PAD, we assessed statin prescription and LDL-baseline concentrations at the time of first peripheral arterial intervention, stratified for patients with and without polyvascular disease, as well as the influence of polyvascular attainment on mortality and cardiovascular outcomes.

We here report four major findings: (i) PAD patients with additional manifest polyvascular atherosclerotic disease are older, have a higher prevalence of cardiovascular risk factors and more impairment of renal function; (ii) statin treatment is more common and more intense in polyvascular PAD patients to improved LDL-C target attainment than in PAD-only patients; (iii) despite more favorable LDL-C management, all-cause and cardiovascular mortality was higher in polyvascular PAD patients; (iv) +2 V-polyvascular disease had the strongest association with overall mortality.

Previous reports considered only parts of our data set. Our findings are in line with some parts of previous studies: a secondary analysis of the EUCLID trial showed similar proportions of polyvascular patients and outcomes in polyvascular patients compared to PAD alone, as well as an association of polyvascular disease with mortality and CVE [16]. We here report some novel findings: polyvascular patients were prescribed higher doses of statins than patients with PAD alone, which may be due to their more regular need for physician visits as well as due to their more extensive history of the disease and the physician’s and patient’s stronger attention for the need of excellent risk factor management. In previous studies, PAD patients were less likely to have guideline conform lipid-lowering therapy [21,22,23].

Our multivariable model revealed that factors influencing overall mortality were polyvascular disease, age, and impaired kidney function; these are similar findings to those in previous studies [1,15,16].

It remains of note that current smoking and high LDL-C at baseline were not associated with elevated mortality risk (LDL-C HR 0.73, 95% CI: 0.55–0.97, active smoker HR 0.90, 95% CI: 0.65–1.24, former smoker HR 0.76, 95% CI:0.52–1.10 respectively) while both are clearly established cardiovascular risk factors. This finding is, with regard to our study population, possibly best interpreted as a reverse epidemiology effect, a phenomenon previously described in elderly patients [24,25], but might also be explained by the fact that the older, polyvascular patients (both shown as independent risk factors) in our study had lower LDL-C values through a more intensive statin treatment, as shown in Figure 2 and Figure 3. Smoking was assessed by questionnaire only, as usual in clinical practice. We did not have data available on nicotine metabolites, e.g., cotinine. Therefore, we cannot exclude the possibility of mal-compliance in answering the questionnaire. Another potential explanation for these findings could be collider bias, considering patients who smoke and have a higher risk of PAD are more likely to be present in the database. Furthermore, diabetes in this population was more frequent. Thus, the lower LDL-C values might also be interpreted as small-dense LDL-particles (sdLDL-C), a form of LDL-C typical in diabetic patients [26], being even more pro-atherogenic and associated with a higher cardiovascular risk [27].

It is established that PAD patients are a population of very high cardiovascular risk and may be of the highest risk when compared to patients with CAD or CeVD [3,4,5]. This risk, especially for (MACE), further increases in the case of a polyvascular extent, as has been shown in randomized trials of long-term secondary prevention [28,29]. When the risk is adjusted for baseline differences, the risk for polyvascular patients even further increases, and this also translates into an even greater absolute risk reduction when the treatment is intensified. Therefore, PAD patients, especially polyvascular patients, appear to be a population with the most favorable risk/benefit ratio [28,29].

Indeed, it has been shown by Bonaca et al. from the FOURIER trial that PAD patients have the highest absolute risk reduction by the PCSK-9 inhibitor Evolocumab and thus the lowest number-needed-to-treat (NNT) [30]. From the ODYSSEY OUTCOMES trial, we have learned that the outcome of the PCSK9 inhibitor arm versus the control arm differs much more widely in patients with polyvascular than in those with monovascular or bivascular disease [31]. These studies reveal that PAD patients show a large heterogeneity of cardiovascular risk.

In the present study, polyvascular patients show a two- to three-fold increase in all-cause mortality when all three vascular regions are affected, despite a more intense treatment at baseline. This shows that PAD itself is not one simple high-risk condition but varies broadly with regard to age, comorbidities (e.g., diabetes and impaired renal function), and particularly atherosclerotic extent.

Therefore, customized therapeutic interventions could further improve NNTs, improve cost-effectiveness, and ultimately stimulate specifically designed outcome trials. Furthermore, our findings should encourage an intensified intervention at the earliest possible time point for patients who are still in a PAD-only state in order to prevent a deleterious aggravation towards a more generalized form of atherosclerosis. Despite the growing body of literature identifying the polyvascular phenotype as a malignant condition with extremely high cardiovascular risk, little is known about the progression of patients with PAD alone toward a polyvascular disease. Results from a prospective observational study with 109 claudicants (Fontaine stage IIa) showed that an aggravation towards a polyvascular disease occurred in over 60%, with 20% in all three vascular beds [32]. Mortality was similarly high in our population at 26%, including 39% deaths from vascular causes. Conveying this onto our population with 688 PAD-only patients, one might expect over 400 patients to develop a polyvascular disease, with approximately 140 patients having an aggravation towards two further vascular beds. Keeping in mind that in our population of PAD-only patients, most patients were undertreated regarding lipid-lowering therapy and LDL-C goal attainment, this calculation is not farfetched.

## 5. Limitations

A limitation of this study is the single-center study design and the retrospective nature. Second, data on cardiovascular events may be incomplete: we may have missed events of patients that could have moved to other hospitals after the completion of our endpoint analysis. Third, potential confounding factors such as inflammation, presence of other comorbidities, or nicotine metabolites were not captured. The patients enrolled in this study are the most severe patients from a tertiary reference center, and patients with TG > 4.5 mmol/L were excluded from this analysis; thus, the findings may not be generalizable to other patient populations. Fourth, considering almost 25% of patients were not included in our analysis due to missing lipid values at baseline or exclusion for high TG, selection bias could be present. This could be further confirmed by the results of Kaplan–Meier showing that the differences between the groups were present within the first days of follow-up, which could be an indication of inherent bias and differences in population characteristics between the PAD groups. Finally, confounding by indication and collider bias could also be present, which due to the nature of the retrospective design, were not possible to be explored with regard to the outcome of the study.

## 6. Strengths

This study is a large real-world registry of patients undergoing their first peripheral arterial intervention. Follow-up information of almost 96% of those patients included in the analysis is available.

## 7. Conclusions

Identifying undertreated PAD patients with a risk for progression into polyvascular disease and designing randomized trials in secondary prevention powered for the PAD subpopulation should be a goal for the future to develop more specific, customized interventions. However, those already identified need to be treated intensively according to present guidelines. Future studies are needed to explore whether more aggressive LDL-lowering treatment for PAD patients may be translated into better prognosis.

## Figures and Tables

**Table 1:** Baseline demographics.

Demographics	All Patients n = 1377	PAD only n = 688	+1 Vessel n = 552	+2 Vessel n = 137	*p*-Value
Age (years), mean ± SD	72.0 ± 11.7	70.2 ± 12.4	73.2 ± 11.0	75.6 ± 8.9	<0.0001
<55 years, n (%)	107 (7)	80 (12)	27 (5)	0 (0)	<0.0001
55–75 years, n (%)	655 (48)	336 (49)	261 (47)	58 (42)	n.s.
>75 years, n (%)	615 (45)	272 (39)	264 (48)	79 (58)	<0.0001
Female gender, n (%)	501 (36)	281 (41)	181 (33)	39 (28)	0.002
CVD family history, n (%)	178 (13)	70 (10)	84 (15)	24 (18)	<0.008
Current smoker, n (%)	635 (46)	363 (53)	226 (41)	46 (34)	<0.0001
Diabetes, n (%)	419 (30)	165 (24)	194 (35)	60 (44)	<0.0001
HbA1c, % median (Q1, Q3)	6 (5.7, 6.7)	6 (5.6, 6.5)	6.1 (5.7, 6.9)	6.3 (5.8, 7.1)	<0.0001
Hypertension, n (%)	1179 (86)	539 (78)	507 (92)	133 (97)	<0.0001
Total Cholesterol (mmol/L) median (Q1, Q3)	4.3 (5.6, 5.1)	3.7 (4.6, 5.3)	4.1 (3.5, 4.8)	3.9 (3.3, 4.7)	<0.001
LDL-C (mmol/L) median (Q1, Q3)	2.4 (1.7, 3.1)	2.6 (1.9, 3.4]	2.2 (1.6, 2.8)	2.1 (1.5, 2.7)	<0.0001
HDL-C (mmol/L) median (Q1, Q3)	1.2 (1.0, 1.5)	1.3 (1.0, 1.5)	1.2 (1.0, 1.5)	1.1 (0.9, 1.4)	0.0016
TG (mmol/L) median (Q1, Q3)	1.5 (1.1, 2.2)	1.4 (1.1, 2.1)	1.5 (1.1, 2.2)	1.5 (1.1, 2.2)	0.15
Chronic Kidney disease (CKD), n (%)	955 (69)	429 (62)	416 (75)	110 (80)	<0.0001
Stage 2, n (%)	496 (52)	248 (50)	200 (40)	48 (10)	n.s.
Stage 3a, n (%)	222 (23)	93 (42)	105 (47)	24 (11)	0.03
Stage 3b, n (%)	152 (15)	63 (42]	66 (43)	23 (15)	0.023
Stage 4 and 5, n (%)	85 (7)	25 (5)	45 (8)	15 (11)	<0.0001
Creatinin, µmol/L median (Q1, Q3)	82 (67, 107)	64 (76, 95)	88 (72, 114.5)	95 (73, 128)	<0.0001
Glomerular Filtration Rate (eGFR), (ml/min)	69.1 ± 22.7	73.2 ± 21.5	65.3 ± 23.7	60.9 ± 23.6	<0.0001
Severity of PAD (Fontaine stage)					
Intermittent claudication II, n (%)	799 (58)	399 (58)	329 (60)	71 (52)	0.26
II a, n (%)	186 (23)	95 (24)	76 (23)	15 (21)	0.89
II b, n (%)	613 (77)	304 (76)	253 (77)	56 (79)	
Chronic limb-threatenting ischemia III, n (%)	180 (13)	91 (13)	70 (12)	19 (14)	0.92
IV, n (%)	398 (29)	198 (29)	153 (28)	47 (34)	0.23

**Table 2:** Multivariable cox-regression and analysis of the association of cardiovasvular risk factors, statin use and polyvascular disease vs. PAD only on overall mortality. (a): adjusted for age and sex; (b) adjusted for cardiovascular risk factors and statin use.

	HR (95% CI)	*p*-Value
a		
Age [years]	1.05 (1.04–1.06)	<0.001
Female sex	0.63 (0.47–0.82)	0.004
b		
PAD only	1 (Reference)	
+1 Vessel	1.64 (1.22–2.20)	0.001
+2 Vessel	2.65 (1.82–3.84)	<0.001
Age (years)	1.03 (1.01–1.04)	<0.001
Female sex Hypertension	0.87 (0.57–1.02) 0.90 (0.59–1.40)	n.s. n.s.
Creatinine * (μmol/l)	2.47 (1.94–3.15	<0.001
Never Smoker	(Reference)	
Former smoker	0.76 (0.52–1.10)	n.s.
Active smoker	0.90 (0.65–1.24)	n.s.
Diabetes mellitus	1.30 (0.99–1.70)	n.s.
LDL-C* (mmol/L)	0.73 (0.55–0.97)	n.s.
Statin use	0.79 (0.60–1.05)	n.s.

* variables log-transformed.

## Data Availability

Restrictions apply to the availability of these data. Data was obtained from University Hospital Bern and are available with the permission of University Hospital Bern.

## Supplementary Materials

The following supporting information can be downloaded at: https://www.mdpi.com/article/10.3390/jcm12103410/s1, Figure S1: Study Flow Chart; Figure S2a: Schoenfeld residual plot for proportional hazards assumption (model 1, adjusted for age and sex); Figure S2b: Schoenfeld residual plot for proportional hazards assumption (model 2, adjusted for +1 V, +2 V and cardiovascular risk factors); Figure S3: Kaplan-Meier Curves and log-rank test results of endpoints: (**a**) all-cause mortality, (**b**) cardiovascular mortality and (**c**) cardiovascular event rates for PAD only, +1V- and +2V- patients. Relative distribution of (**d**) all-cause mortality, (**e**) cardiovascular mortality and (**f**) cardiovascular event rates for PAD only, +1V- and +2V- patients; Table S1: Multivariable cox-regression and analysis of the association of cardiovasvular risk factors, statin use and polyvascular disease vs. PAD only on overall mortality, stratified by sex; Table S2: LDL-target categories stratified for Polyvascular extent; Table S3: Multivariable cox-regression and analysis of the association of cardiovasvular risk factors, statin use and polyvascular disease vs. PAD only on cardiovascular events (MACE&MALE).
