# Supplementary material for: Prognostic Role of Polyvascular Involvement in Patients with Symptomatic Peripheral Artery Disease
Source: J Clin Med. 2023 May 11;12(10):3410. doi: 10.3390/jcm12103410 (PMC10219171; doi:10.3390/jcm12103410)
Supplement: Supplementary file 1 [file jcm-12-03410-s001.zip › jcm-2303661-supplementary.pdf]

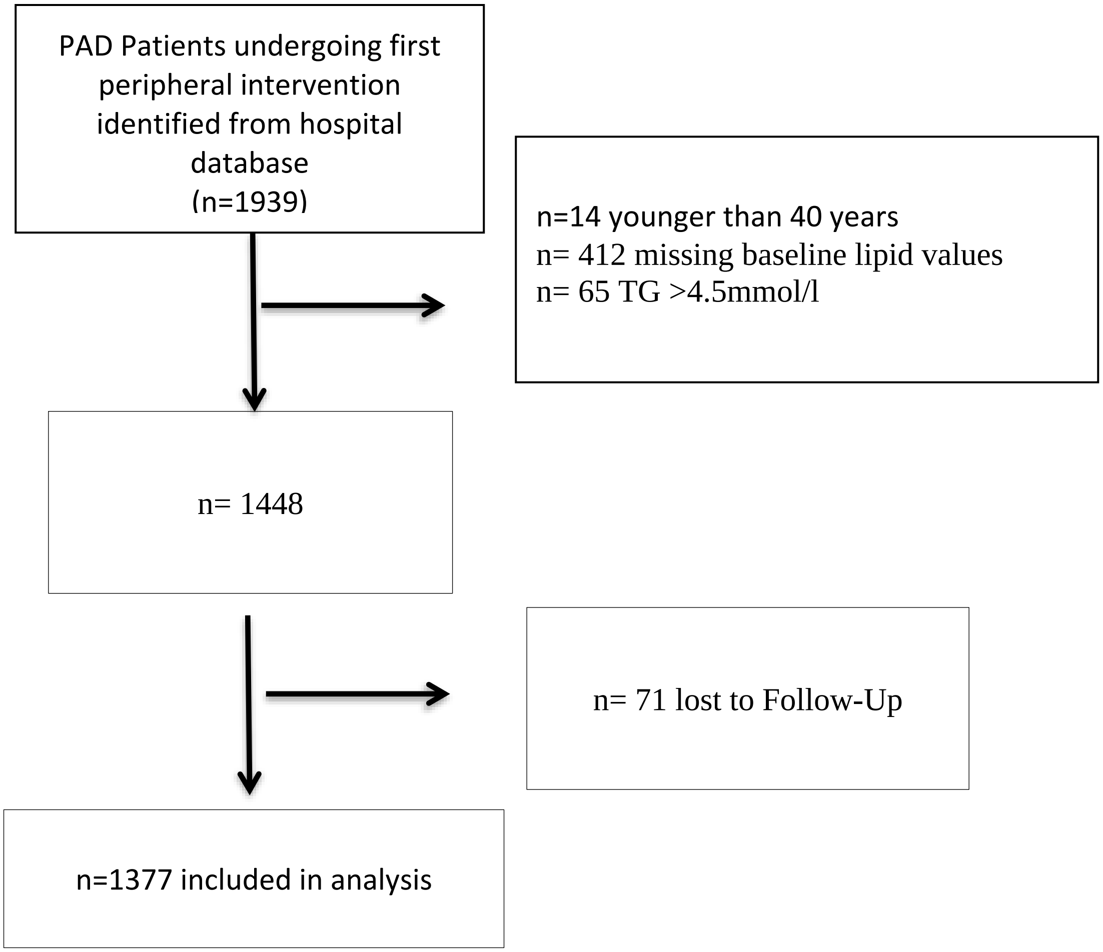

Figure S1: Study Flow Chart.

Table S1: Multivariable cox-regression and analysis of the association of cardiovasvular risk factors, statin use and polyvascular disease vs. PAD only on overall mortality, stratified by sex

	Male		Female	
	**HR (95% CI)**	**p-value**	**HR (95% CI)**	**p-value**
PAD only	1 (Reference)			
+1 Vessel	1.35 (0.93-1.95)	n.s.	2.21 (1.35-3.62)	0.002
+2 Vessel	2.33 (1.49-3.65)	<0.001	3.37 (1.70-6.70)	0.001
Age [years]	1.03 (1.01-1.05)	0.001	1.03 (1.01-1.06)	0.016
Hypertension	0.85 (0.51-1.41)	n.s.	1.04 (0.45-2.44)	n.s.
Creatinine* (μmol/l)	2.24 (1.68-2.99)	<0.001	3.43 (2.07-5.70)	<0.001
Never Smoker	Reference			
Former smoker	0.74 (0.49-1.14)	n.s.	0.64 (0.27-1.51)	n.s.
Current smoker	0.80 (0.55-1.18)	n.s.	1.17 (0.65-2.10)	n.s.
Diabetes mellitus	1.36 (1.00-1.95)	n.s.	1.11 (0.70-1.76)	n.s.
LDL-C*(mmol/l)	0.62 (0.66-0.96)	0.008	0.95 (0.58-1.56)	n.s.
Statin use	0.82 (0.56-1.18)	n.s.	0.77 (0.48-1.23)	n.s.

*variables log-transformed

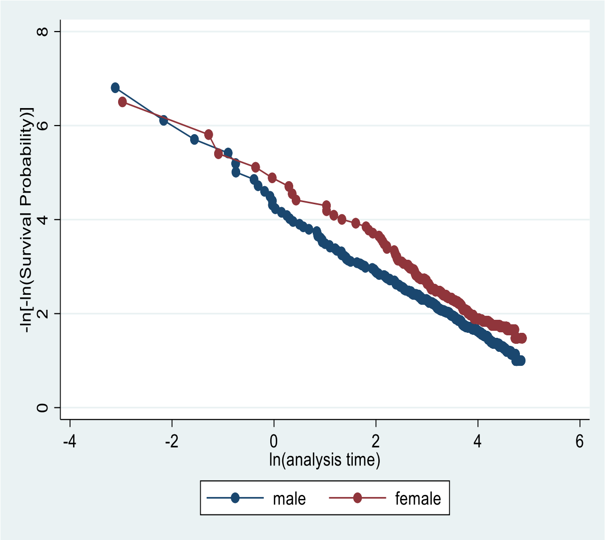

p= 0.1720

Figure S2a: Schoenfeld residual plot for proportional hazards assumption (model 1, adjusted for age and sex).

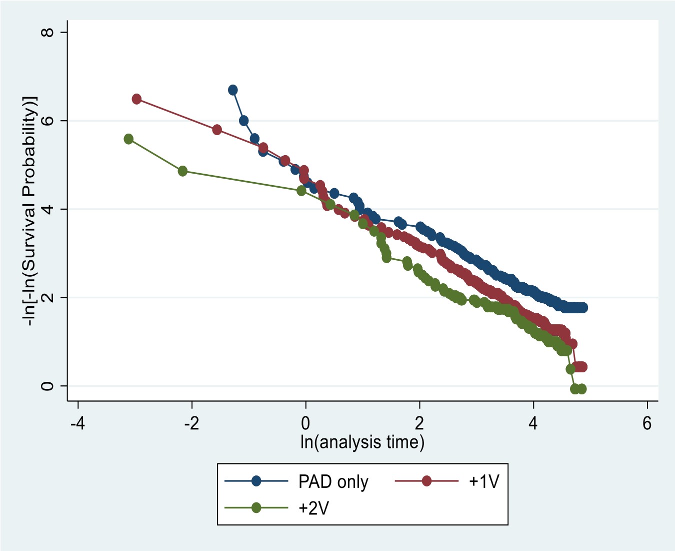

p= 0.0785

Figure S2b: Schoenfeld residual plot for proportional hazards assumption (model 2, adjusted for +1V, +2V and cardiovascular risk factors).

Table S2: LDL-target categories stratified for Polyvascular extent

LDL-C target category	All patients	PAD only	+1V	+2V	p-value
<1.4 n, [%]	178 [13]	68 [38]	86 [48]	24 [14]	0.003
>1.4 <=1.8 n, [%]	200 [14]	78 [39]	90 [45]	32 [16]	<0.001
>1.8 <2.6 n, [%]	438 [32]	204 [46]	192 [44]	42 [10]	n.s.
≥2.6 n, [%]	561 [41]	338 [60]	184 [33]	39 [7]	<0.001

Table S3: Multivariable cox-regression and analysis of the association of cardiovasvular risk factors, statin use and polyvascular disease vs. PAD only on cardiovascular events (MACE&MALE)

Table S3a: adjusted for age and sex

	**HR (95% CI)**	**p-value**
Age [years]	1.00 (0.99-1.02)	n.s.
Female sex	0.68 (0.47-0.98)	0.04

Table S3b: adjusted for cardiovascular risk factors and statin use

	HR (95% CI)	p-value
PAD only	1 (Reference)	
+1 Vessel	1.86 (1.28-2.68)	0.001
+2 Vessel	1.90 (1.10-3.30)	0.021
Age [years]	0.99 (0.98-1.01)	n.s.
Female sex	0.83 (0.56-1.24)	n.s.
Hypertension	1.89 (0.99-3.57)	n.s.
Creatinine* (μmol/l)	1.33 (1.0.86-2.04	n.s.
Never Smoker	(Reference)	
Former smoker	1.27 (0.79-2.10)	n.s.
Active smoker	0.90 (0.58 -1.40)	n.s.
Diabetes mellitus	0.95 (0.66-1.37)	n.s.
LDL-C* (mmol/l)	1.06 (0.71-1.58)	n.s.
Statin use	1.06 (0.71-1.58)	n.s.

*variables log-transformed

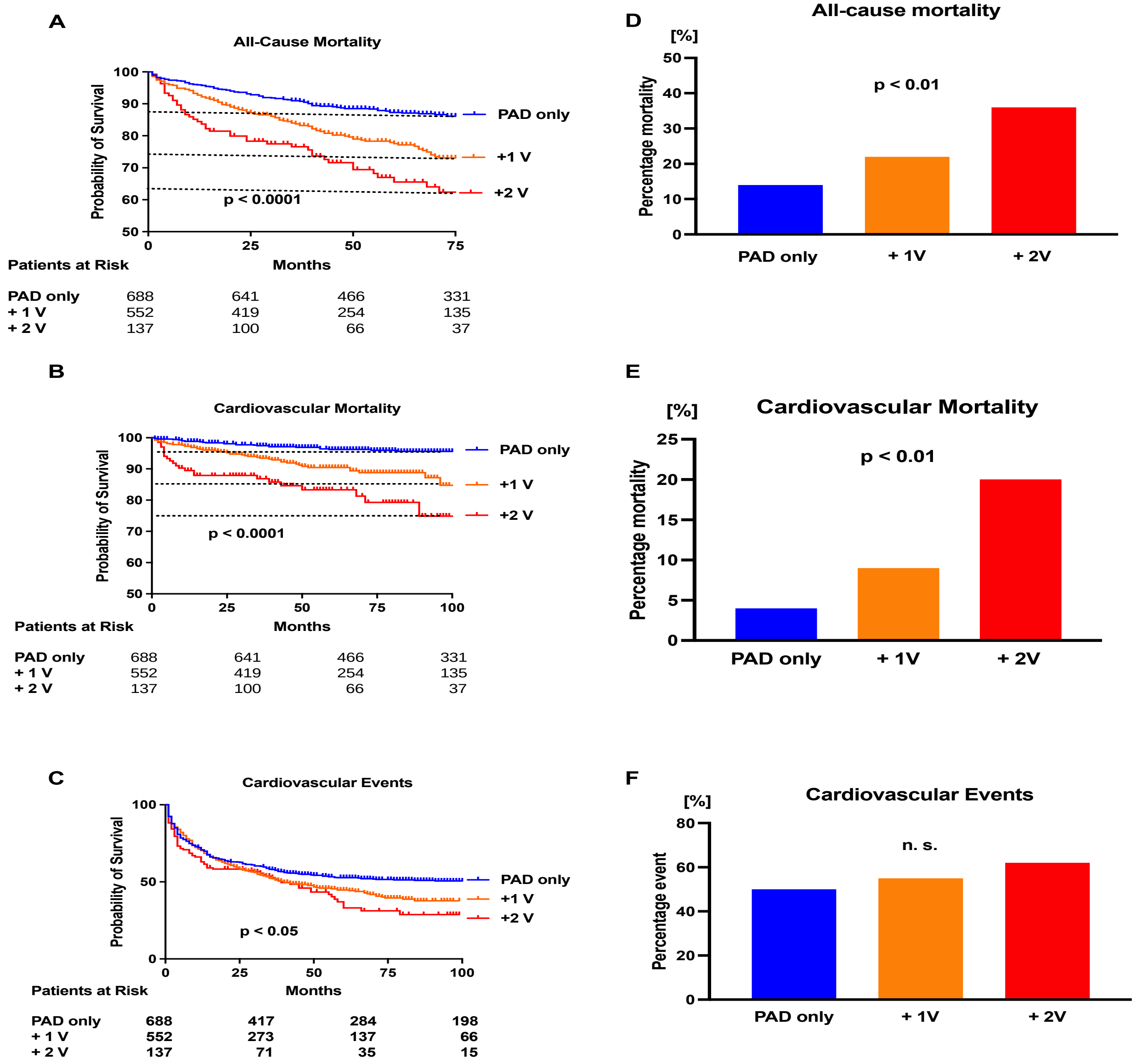

Figure S3: Kaplan-Meier Curves and log-rank test results of endpoints: (a) all-cause mortality, (b) cardiovascular mortality and (c) cardiovascular event rates for PAD only, +1V- and +2V- patients. Relative distribution of (d) all-cause mortality, (e) cardiovascular mortality and (f) cardiovascular event rates for PAD only, +1V- and +2V- patients.
